# Developing an Interprofessional Pediatric Rehabilitation Model of Care in Northern Cree First Nation Communities: Protocol for a Needs Assessment and Codeveloped Intervention With a Qualitative and Participatory Action Approach

**DOI:** 10.2196/69438

**Published:** 2025-09-10

**Authors:** Katie Crockett, Hailey Dunn, Rosalie Dostie, Karin Diedrich-Closson, Laureen McIntyre, Kristen Quigley, Karen Sharpe, Ivar Mendez, Jaris Swidrovich, Rachel Johnson, Veronica McKinney, Tanya Holt, Tami Turner, Karen Bird, Briana Bowes, Carlene Custer, Kiandra Linklater, Chantel Camden, Stacey Lovo

**Affiliations:** 1 School of Rehabilitation Science University of Saskatchewan Saskatoon, SK Canada; 2 School of Rehabilitation Université de Sherbrooke Sherbrooke, QC Canada; 3 Saskatchewan Health Authority Saskatoon, SK Canada; 4 Educational Psychology & Special Education University of Saskatchewan Saskatoon, SK Canada; 5 Developt Pediatric Physical Therapy Saskatoon, SK Canada; 6 Thrive Hearing Solutions Saskatoon, SK Canada; 7 Department of Surgery University of Saskatchewan Saskatoon, SK Canada; 8 Leslie Dan Faculty of Pharmacy University of Toronto Toronto, ON Canada; 9 Angelique Canada Health Centre Pelican Narrows, SK Canada; 10 Northern Medical Services College of Medicine University of Saskatchewan Saskatoon, SK Canada; 11 College of Medicine University of Saskatchewan Saskatoon, SK Canada; 12 Peter Ballantyne Cree Nation Health Services Prince Albert, SK Canada

**Keywords:** telerehabilitation, Indigenous peoples, pediatrics, remote care, rehabiltation

## Abstract

**Background:**

In Canada, the Indigenous population is the youngest and fastest growing, yet ongoing health disparities for Indigenous peoples are widely recognized. There is a concerning lack of research on childhood disabilities and health conditions in Indigenous populations in Canada. For children with disabilities and chronic health conditions, ongoing access to rehabilitation services, such as occupational therapy, physical therapy, speech-language pathology, and audiology, is critical in promoting positive health and developmental outcomes. Elders and the Peter Ballantyne Cree Nation health services board have guided a critical priority for addressing access challenges to pediatric rehabilitation in 3 specific northern Indigenous communities.

**Objective:**

The purpose of this manuscript is to outline the protocol for a community-directed needs assessment and subsequent development of a multidisciplinary pediatric rehabilitation service in 3 specific northern Indigenous communities.

**Methods:**

The study involves 3 phases. In phase 1, the needs assessment process was led by 2 physiotherapy clinician researchers and 2 graduate students working with health care professionals in pediatric speech-language pathology, audiology, physiotherapy, and occupational therapy with experience in both private and public health entities. The process consisted of multiple parts, which included a community-led request, a preliminary literature review, survey development, interview guide development, communication and feedback with health care professionals, a test phase with pediatric family members, and finalization of the survey and interview guides for deployment. In phase 2, the findings from phase 1 will inform the codevelopment of a pilot hybrid-care, interprofessional pediatric rehabilitation clinic for each of the communities. In phase 3, a stakeholder meeting will take place to facilitate knowledge sharing and open discussion regarding the implementation of phase 2 as well as considerations for the sustainability of this model of care.

**Results:**

The final survey was multidisciplinary, with 6 content areas covered in 15 items. Guides for 1-on-1 interviews and sharing circles included 10 questions for community members and 12 questions for health care providers. Participant recruitment began in April 2024. Final results are anticipated in early 2026.

**Conclusions:**

This manuscript details the process of a community-directed needs assessment, which will inform the development and implementation of a model of care for pediatric rehabilitation services. Our process was driven by a request from the community for a needs assessment and emphasized the involvement of key stakeholders early and often during assessment development. A clear purpose of the project was identified with community direction. We used multidisciplinary inputs from both public and private sectors and maintained clear goals during our survey question design process. This study aims to inform the codevelopment and implementation of an interdisciplinary, hybrid model of pediatric rehabilitation care for remote First Nation communities, ultimately leading to improved access to patient- and family-centered care for pediatric rehabilitation.

**International Registered Report Identifier (IRRID):**

DERR1-10.2196/69438

## Introduction

In Canada, the Indigenous population is the youngest and fastest growing [1], yet there is evidence for ongoing health disparities [2-4]. Within the scope of this paper, Indigenous refers to all of First Nations, Métis, and Inuit peoples. There is a concerning lack of research to provide accurate data on childhood disabilities and health conditions in Indigenous populations in Canada [2,4]; however, based on a community-driven request and community knowledge, there is a growing need for rehabilitation services for pediatric families in specific northern Indigenous communities in Saskatchewan. Previous research conducted in Saskatchewan has identified barriers to rehabilitation care, impacts on family and quality of life, and recommendations for improving services for rehabilitation in an Indigenous community [5].

In 2007, Jordan’s Principle was created to ensure that Indigenous children had appropriate access to health care [4]; despite this, they are still not able to appropriately access the care they need [2,6]. In 2016, the Canadian Human Rights Tribunal highlighted important shortcomings regarding early intervention and family programs offered to Indigenous peoples and concluded that the Canadian government was systematically discriminating against Indigenous peoples [7]. Beyond the specific challenges associated with Jordan’s Principle, children in rural and remote areas, including Indigenous children, face significant additional barriers to access services. These include incredibly long wait times and the necessity for families to travel, which brings associated challenges, such as appropriate transportation, adverse weather conditions, financial costs, time constraints, and the need for childcare for other children in the family [5,8,9]. Indigenous children often encounter additional health disparities rooted in historical, social, and economic factors that necessitate a holistic and culturally sensitive approach to rehabilitation [6].

For children with disabilities and chronic health conditions, ongoing access to rehabilitation services, such as occupational therapy (OT), physical therapy (PT), speech-language pathology (SLP), and audiology (AUD), is critical for promoting positive health and developmental outcomes [10]. Interprofessional collaboration among these disciplines is considered to promote best practice in rehabilitation as it can enhance efficiency, patient outcomes, and clinician and patient satisfaction [11]. In Indigenous communities, the implementation of interdisciplinary rehabilitation for pediatrics is imperative due to the unique challenges faced by children and their families in these populations. Interdisciplinary rehabilitation, involving collaboration among health care professionals in PT, OT, SLP, and other disciplines with guidance and teaching by community experts is essential to address the multifaceted needs of Indigenous pediatric patients and their families. By integrating diverse expertise, practitioners can tailor interventions to consider cultural contexts, traditional healing practices, and community dynamics. This approach not only enhances the effectiveness of rehabilitation but also fosters a comprehensive understanding of the child’s well-being within the broader cultural and familial context, ultimately contributing to more meaningful, culturally responsive, and sustainable health outcomes for Indigenous pediatric populations and their families.

The purpose of this manuscript is to outline the protocol for a community-directed needs assessment and subsequent development of a multidisciplinary pediatric rehabilitation service in 3 specific northern Indigenous communities. The research uses a single group, with a participatory action approach, following an ethical space and two-eyed seeing framework [12-14]. The protocol follows an identified critical priority area guided by Elders and the Peter Ballantyne Cree Nation (PBCN) health services board in order to address access challenges to pediatric rehabilitation in 3 specific northern Indigenous communities. The protocol includes a final critical phase for a multistakeholder meeting to facilitate knowledge sharing and open discussion.

## Methods

### Ethical Considerations

This research has been approved by the University of Saskatchewan Behavioural Research Ethics Board (REB, #3486). Protocol amendments will be approved by the REB. Each participant will be provided with a consent form approved by the REB (Multimedia Appendix 1) with an opportunity to review the research project details with a research team member or community research assistant (CRA) verbally to ensure complete understanding and informed consent. Translators will be provided as needed to ensure understanding. All data will be deidentified. Complete anonymity will not be possible due to the nature of the sharing circles. Patient participants will receive honorarium for their participation following the University of Saskatchewan protocol for research honorarium. All data will be held by the principal investigator in a secure, password-protected, web-based data platform approved by the University and REB. We will request input from patients and sharing groups as to how they would like to find out about the results of the study and ensure that the preparation of desired methods occurs at the direction of the community.

This needs assessment and plan to develop a pilot hybrid, interprofessional care model for remote and in-person pediatric rehabilitation was based on a community-identified need and request. The request came from an Elder’s Advisory Council and was based on successful research projects involving remote models of care for adult populations [15,16]. The council and the PBCN health services board identified the specific communities in need. Eligible participants were recruited from these communities with self-reported pediatric rehabilitation needs across any one or combination of disciplines (PT, OT, SLP, or AUD). Eligible health care participants were any health care provider, service coordinator, or administrator serving at least one of the identified communities. A patient-oriented research approach was used following ownership, control, access, and possession principles and adhering to chapter 9 of Tri-Council policy statement 2 on research involving First Nations, Inuit, and Métis peoples of Canada [17-19]. Our diverse multidisciplinary research team includes patient partners, respected Elders, and Knowledge Keepers, with support from local community health directors and Jordan’s Principle experts. Our research team has experience in Indigenous health, health services research, collaborative models of care, musculoskeletal health, mixed methods, rural and remote health service delivery, community-based participatory action health research approaches, virtual health, and Jordan’s Principle. Team members VM and SL have over 15 years and 10 years, respectively, of collaborative relationships with the Pelican Narrows Elders and PBCN.

This research was grounded in community-based participatory action research, a two-eyed seeing and ethical space framework [12-14]. Community-based participatory action research emphasizes collaboration between researchers and all stakeholders throughout the entirety of the research process—beginning from developing the research questions through to data collection, analysis, and dissemination of the findings [13]. This process ensures that the research is based on the needs and concerns of the community involved and supports or enhances strategic action in resolving community issues while maintaining an emphasis on invaluable community knowledge and equal inclusion and collaboration [13].

### Phase 1: Needs Assessment and Relationship Building

#### Overview

Phase 1 included relationship building, planning, developing sharing circle guides, and trips to participating communities for needs assessment data gathering (sharing circles and semistructured interviews when requested, sharing of meals, and gifting to community members) to identify community and family strengths; cultural needs, preferences and considerations; as well as barriers to the provision of interprofessional telerehabilitation services.

**Figure 1:**
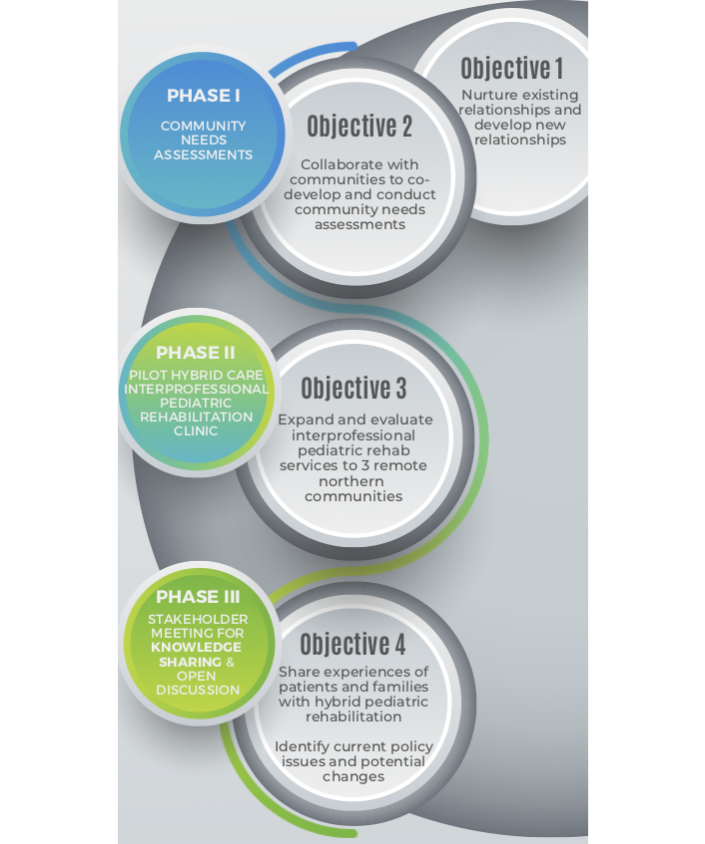
Schematic of the protocol.

**Figure 2:**
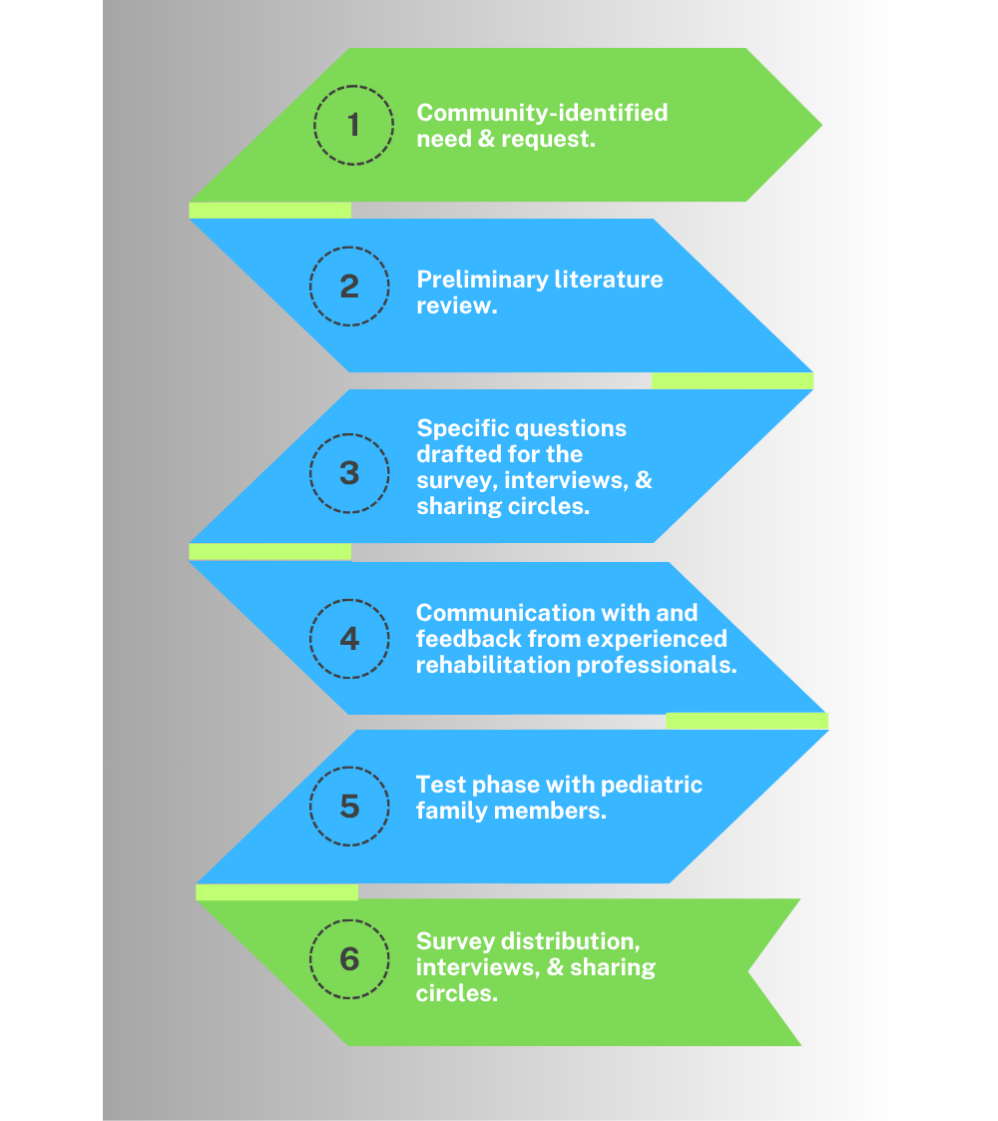
Outline of the needs assessment process.

The needs assessment process was led by 2 physiotherapy clinician researchers and 2 graduate students who worked with health care professionals in pediatric SLP, PT, OT, and AUD with experience in both private and public health entities and collaboration with Indigenous communities. The process consisted of multiple phases, which included a community-led request, preliminary literature review and scoping review [20], survey development, interview guide development, communication and feedback with health care professionals, a test phase with pediatric family members, and finalization of the survey and interview guides for deployment for data collection. The interview guide and survey were developed with community Elders and persons with lived experience. Schematics of the protocol and needs assessment process are outlined in Figures 1 and 2, respectively. CRAs will identify and liaise with potential community members and Elders to participate in the recruitment of family participants, data collection, and organization of semistructured interviews and sharing circles. The needs assessment will include 8-10 families with children with neurodevelopmental or orthopedic rehabilitation needs in each community as well as 2-3 Elders and 2-3 health care providers identified through consultation with the PBCN health services board and patient team members and supporters. Translation will be provided to ensure inclusion of those who are interested. All participants will reside within the 3 identified remote communities and have identified pediatric rehabilitation needs.

#### Web-Based Survey

A demographic and general health questionnaire (Multimedia Appendix 2) will be completed before interviews or sharing circles take place. The web-based questionnaire will be filled out using the research electronic data capture (REDCap) web application [21,22] with an option for a paper questionnaire based on preference. Assistance will be provided by our research assistants or student researchers. A completion check will be performed prior to the interview.

#### Interviews and Sharing Circles

The use of sharing circles as an Indigenous methodology in research allows participants to safely share stories [23,24]. Participants will be provided the option for an individual interview or participation in a sharing circle. Up to 20 individual interviews and 1-3 sharing circles will take place. The final sample size will be determined by the emerging analysis; however, we anticipate this estimated sample size is reasonable to reach data saturation based on our team’s previous work in similar qualitative analyses. Interviews or sharing circles will be held in person or remotely using a videoconference platform (Zoom) or the remote presence robot in the community health center, based on participant preference. An approved interview guide will be used (Appendices 3 and 4). Interviews will be recorded, transcribed using Otter.ai, and analyzed thematically using qualitative coding software (NVivo, QSR International). Participants will have the opportunity to review transcripts prior to data analysis and synthesis. Interview and sharing circle findings will be examined together with the research team, including patient advisor team members, during the analysis phase to guide the next stage of the project. Analysis will involve a qualitative, interpretive research approach with inductive thematic analysis [25,26]. To support reflexivity, an intersectional approach, and a patient-oriented research approach, the team will meet and collaborate multiple times. This process will ensure that Indigenous worldviews, language, culture, community practices, and protocols continue to guide the research.

### Phase 2: Pilot Hybrid-Care, Interprofessional Pediatric Rehabilitation Clinic

Phase 2 will be informed by the findings from phase 1 and will feature codevelopment and implementation of a pilot hybrid-care, interprofessional pediatric rehabilitation clinic in each community using a combination of remote presence technology (RPR) and in-person care. For example, the types of care provision and combinations of services needed and the complexity of care will be revealed through the survey responses, further defining the community’s needs. Patient experiences and needs will be further understood through qualitative analyses, followed by deliberation with patient partners, health care providers, community partners, and Knowledge Keepers to codevelop and implement a pilot hybrid-care pediatric rehabilitation clinic.

Planning will occur in collaboration with community families and Elders. CRAs will bring Indigenous community protocols, practices, and knowledge to our team approach, which is essential to guiding academic team members in respectful community engagement. It is anticipated that the team will comprise urban-based PT, OT, SLP, and AUD providers in addition to the CRAs who will be physically present. CRAs will receive formal training by team partners in research protocols and team functioning and will be supported through RPR technology as well as Zoom and email.

The urban-based rehabilitation providers will join remote families and their local primary care providers for assessment using RPR and provide follow-up care through remote and in-person sessions, as determined by the practitioner. It is anticipated that in-person sessions will be required for some components of assessment and care provision. A total of 15 participants from each of the 3 communities will participate in the assessment and 2 additional follow-up treatments (at least one of which will be in person).

Demographic, functional, and cost outcomes will comprise the quantitative data. Outcome measures will be developed in partnership with Elders, patient team members, and persons with lived experience to ensure community direction in the evaluation and may include quality of life and pediatric functional and cognitive measures. Family, community, and provider stories and experiences will provide qualitative information. Following participation in the hybrid interprofessional care clinic, pre- and posttest data collection by researchers and in partnership with the CRA will be quantitatively analyzed. Follow-up, semistructured interviews with participants will be audio recorded and transcribed using Otter.ai. Qualitative analysis will proceed as described earlier in phase 1. All participants will receive honorarium for their participation.

### Phase 3: Stakeholder Meeting for Knowledge Sharing and Open Discussion

This phase will include a stakeholder meeting where food and reciprocal knowledge will be shared and open discussion will occur to review experiences and findings from the needs assessments and interprofessional pediatric rehabilitation clinics. This process will be developed specifically with the PBCN health services board and Jordan’s Principle experts so the context is correct, as recommended by Smylie et al [27]. Families will be invited to share their stories. System and jurisdictional issues will be discussed, and idea generation from unique stakeholder perspectives will be used to develop considerations and recommendations to ensure sustainability and encourage uptake of the model locally and across other Indigenous communities. Participants will include families and community members, PBCN health services board and community Chief and Councils, education sectors, provincial and federal health governance administrators, researchers, and health care providers.

## Results

The final survey was multidisciplinary, with 6 content areas covered in 15 items. Content covered in the web-based survey included the following categories: about your child, about your child’s birth and development, about your child’s health history, use of equipment, more about your child (eg, strengths and favorite activities and toys), and about your family (Multimedia Appendix 2). Interview guides for community members included 10 questions, with a focus on allowing space for participants to share about their child and the conditions requiring rehabilitation services, how this has affected the child’s and family’s life, experiences in accessing rehabilitation services, and opinions on using remote technology with the support of local health care providers (Multimedia Appendix 3). Interview guides for health care providers included 12 questions, with a focus on allowing space for participants to share broadly about their observations regarding children and families needing pediatric rehabilitation services in the community. These included barriers and facilitators for children and their families to access appropriate services and supports, opinions on using remote technology with the support of local health care providers, as well as culturally safe practices and pediatric outcome measures (Multimedia Appendix 4).

The results of the needs assessment survey, interviews, and sharing circles will inform the development and implementation of the multidisciplinary hybrid model of care, using both in-person and RPR sessions. The results of the pilot model of care will provide information on multidisciplinary needs of families and children and a collaborative approach to rehabilitation care. We anticipate learning of further gaps in care and improvements to the model, including efficiencies across disciplines, information and resource sharing implications, and other logistics that may impact policy and decision-making for funding considerations. Finally, the results of the community engagement stakeholder meeting will provide critical information on future research directions to inform rehabilitation models of care and policy implications for sustainability of the model locally and across other Indigenous communities.

This protocol paper was first submitted in November of 2024. Since that time, we have completed the initial data collection from 22 pediatric patients from 20 different families, as some families had more than 1 child with rehabilitation needs. We have completed interviews with 4 health care providers for the phase 1 needs assessment. Our team travelled to 1 community in 2023, 1 in May 2024, and 1 in April 2025, with another trip planned to the first community in September 2025. We have had ongoing communication with all 3 communities throughout the needs assessment phase as well as regular research team meetings. It is anticipated that the 3 codeveloped hybrid clinics will be fully operational between June and September of 2025. The phase 2 evaluation is anticipated to take place early 2026, with the phase 3 stakeholder gathering anticipated for the spring of 2026.

## Discussion

### Anticipated Findings

Based on advisement from Elders and the community, it is anticipated that study’s findings will reveal unmet needs for pediatric rehabilitation services. We anticipate complex care needs in many cases, where the services needed will include PT, OT, SLP, AUD or a variety of combinations of multiple services. We anticipate pediatric and family desires for a hybrid in-person and remote care model. The codevelopment of services will likely be complex and varied across the communities based on variations in current strengths and gaps in care, with recommendations for further improvements after implementation. The study is also expected to reveal process considerations to ensure effective integration into the existing, strong local health care system. It is anticipated that a hybrid model of care will improve access to needed services in these communities.

This research project is positioned to contribute significantly to our understanding of remote Cree community pediatric rehabilitation needs, gaps in care, and community preferences for the provision of services. The anticipated development of a community-led rehabilitation model of care that is culturally responsive and community-specific aligns with the principles of community-based participatory action research and the two-eyed seeing framework. Two-eyed seeing is a research framework that combines knowledge of both Western and Indigenous worldviews. Although it is not a local teaching, this framework was chosen by our team to serve as a reminder to equally consider and integrate the viewpoints of both Indigenous and Western ways of knowing in this research while prioritizing Indigenous knowledge and experiences. By engaging patients and stakeholders, the project aims to build capacity for future patient-oriented research endeavors, reflecting a commitment to inclusive and respectful practices.

Evidence for the use of telerehabilitation or remote care models to provide care to Indigenous children and their families is scarce. Through a scoping review of the literature, only 3 articles investigating remote care model interventions were identified [20]. Only 1 study was conducted in the Canadian context and explored the feasibility of conducting speech and language assessments of Indigenous children through videoconferencing [28]. Seven children aged 4 to 13 years were evaluated using various tools both onsite and offsite by different SLPs. Results from that study were promising, as the agreement was high for some tests (eg, receptive language and vocabulary abilities) and children responded well to the offsite evaluator. The authors highlighted the importance of in-person visits to ensure cultural responsiveness in the provision of services and develop and nurture relationships and specified that an assistant located in the community is essential to reduce potential evaluation bias and errors in scoring, entertain the child, and handle technological difficulties [28].

A recent systematic review suggests that telehealth is a promising solution for families with children with multiple diagnoses and chronic conditions or developmental disabilities. There is strong evidence for the use of telehealth as an efficient method to improve access to services, decrease waiting times, and improve child-related outcomes in a way that is acceptable for parents [29-32]. Family-oriented services, like coaching aimed at parental support, knowledge transfer, and interventions targeting parental capacity building, seem to be more efficient when delivered remotely [30]. However, for family-centered interventions to have meaningful impacts on Indigenous families’ lives, they must be anchored in Indigenous knowledge, principles, and visions of child development. Such interventions have the potential to provide tools and support to parents and families.

Cultural worldviews, lived experiences, and the influence of language impact the experiences of Indigenous peoples with health systems and health care. Unique community strengths can contribute to the well-being of individuals and children, providing them with a safe environment to learn and grow, and promotes health sovereignty in communities. Methods and systems of health care that work in urban areas are not necessarily appropriate for remote areas, and Western systems and models are not necessarily culturally responsive or appropriate for remote Indigenous communities.

Community needs and cultural safety in practice will vary between urban and rural or northern services and across Indigenous communities whose histories, resources, and resiliencies are different [33]. This has been demonstrated with Indigenous children with presumed or diagnosed neurodevelopmental challenges and their families in rural, isolated, or northern communities; however, there is very little practical guidance for Western-trained practitioners to engage in culturally safe pediatric rehabilitation practices within Indigenous communities in Canada [33]. This project is community-led, with engagement from multiple groups and knowledge users, including patient team members and interdisciplinary rehabilitation experts with extensive experience in pediatric rehabilitation across public and private health entities.

### Strengths and Limitations

Given the dearth of evidence on rehabilitation models of care for Indigenous pediatric patients and their families, this collaborative research will inform future models of care that address community needs, improved accessibility to rehabilitation services, and communities’ perspectives on RPR technology. The project is designed to be community-led, gathering both patient and health care provider perspectives, in order to address barriers to accessing care while realizing current strengths within each community. The research will be conducted using an ethical space framework emphasizing the creation of space for dialogue and collaboration where diverse worldviews and knowledge systems are acknowledged and respected with the Cree philosophy *wahkohtowin*, based on kinship and the importance of relationship-building throughout the process [12]. The final phase allows for further community input during and after the intervention is designed and implemented.

The limitations of this research include the focus on 3 specific communities in Saskatchewan, which may not allow for direct generalizability across other communities. The participating communities use the Cree TH dialect, which may not be generalizable to the languages and worldviews of other Indigenous communities. We anticipate challenges in predicting weather or environmental occurrences (eg, wildfires) for clinical travel requirements to the communities, which may affect the ability to provide in-person services at different times seasonally. These limitations will affect the provision of services and potentially increase reliance on remote services during these periods.

### Future Directions

This research project aims to uncover and understand pediatric rehabilitation needs in northern remote First Nation communities while positioning itself as a catalyst for positive change in rehabilitation practices. By combining community-led approaches, engagement with diverse stakeholders, and a commitment to cultural responsiveness, the project has the potential to make meaningful contributions to the field of pediatric rehabilitation, especially within Indigenous communities in Canada. Future directions will include continual improvement of the model with the addition of future team members identified as needs during the intervention and expansion to other First Nation and Métis communities in Saskatchewan. Dissemination components will be determined through collaboration with community experts and patient partners. This may include culturally specific and relevant language for the development of appropriate pediatric rehabilitation education materials.

## Appendix

Multimedia Appendix 1 Consent form.

Multimedia Appendix 2 Survey.

Multimedia Appendix 3 Interview guide for patients and families.

Multimedia Appendix 4 Interview guide for health care providers.

## Abbreviations

AUD: audiology
CRA: community research assistant
OT: occupational therapy
PBCN: Peter Ballantyne Cree Nation
PT: physical therapy
REB: Research Ethics Board
REDCap: research electronic data capture
SLP: speech-language pathology
RPR: remote presence technology

## Data Availability

Datasets generated and analyzed for this research can be requested by contacting the principal investigator at stacey.lovo@usask.ca and will be provided upon reasonable request. Discussion with the First Nation community would need to occur in keeping with the principles of ownership, control, access, and possession of data for First Nations community-led research.
