# Supplementary material for: Developing an Interprofessional Pediatric Rehabilitation Model of Care in Northern Cree First Nation Communities: Protocol for a Needs Assessment and Codeveloped Intervention With a Qualitative and Participatory Action Approach
Source: JMIR Res Protoc. 2025 Sep 10;14:e69438. doi: 10.2196/69438 (PMC12461169; doi:10.2196/69438)
Supplement: Multimedia Appendix 1 [file resprot_v14i1e69438_app1.docx]

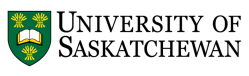


***Participant Consent Form: Individual Interviews***

**You are invited to participate in a research study entitled:** Expanding Interprofessional Pediatric Rehabilitation in Northern Indigenous Communities with a Community-Directed, Team and Virtual Care Approach

**Student Researcher(s):** Hailey Dunn, PhD. Student, College of Medicine, University of Saskatchewan, hab291@usask.ca

Rosalie Dostie, PhD. Student, Faculty of Medicine, Universite de Sherbrooke, Rosalie.Dostie@USherbrooke.ca

**Researcher(s):** Jaris Swidrovich, Assistant Professor, Leslie Dan Faculty of Pharmacy University of Toronto, jaris.swidrovich@utoronto.ca

Ivar Mendez, Director of Virtual Health Hub, Saskatchewan Indian Institute of Technologies and University of Saskatchewan, ivar.mendez@usask.ca

Veronica McKinney, Director of Northern Medical Services, mckinney.nms@usask.ca

Chantal Camden, Professeur-chercheur, École de réadaptation, Faculté de médecine et des sciences de la santé de l'Université de Sherbrooke , chantal.camden@usherbrooke.ca

Laureen McIntyre, Associate Professor, Department of Educational Psychology and Special Education, University of Saskatchewan, laureen.mcintyre@usask.ca, (306) 966-5266

Tanya Holt, Associate Professor Pediatric Critical Care, University of Saskatchewan, tanya.holt@usask.ca, (306) 844-1068

Katie Crockett, Research Assistant, Adjunct Faculty, School of Rehabilitation Science, University of Saskatchewan, katie.crockett@usask.ca

**Principal Investigator/Supervisor:** Stacey Lovo, BScPT, MSc, PhD, Assistant Professor, School of Rehabilitation Science, University of Saskatchewan, [stacey.lovo@usask.ca](mailto:stacey.lovo@usask.ca), (306) 966-8738

**Purpose and Objective of the Research:**

The purpose of this study is to learn about individual and community needs and preferences for the provision of multi-disciplinary pediatric rehabilitation services in your community. In partnership with your community, we have co-designed a community needs assessment to help us learn about family’s and children’s experiences with accessing pediatric rehab services, including where travel is necessary as well as impressions on the use of technology (remote robot) to complete services. Further, we want to learn how pediatric rehab services can be provided in a culturally appropriate and meaningful way. We want to use the findings to inform later phases of the study which will involve working with community to develop a pilot pediatric rehabilitation clinic with in-person and virtual sessions using remote presence robotics (RPR), with culturally responsive outcome measures appropriate for your community.

**Procedures:**

- Taking part in this study will include a one-on-one interview with researcher team member(s). You will be asked to complete a brief form that explains a bit of detail about yourself, your child, and your family and need for pediatric rehabilitation services. This can be completed online by you in partnership with someone from the research team utilizing REDCap software or on paper if preferred. You will not be identifiable by completing this form, as only your participant ID number will appear on this form. Your participant ID number linked to your name will be stored in a separate Master-list. A link to REDCap’s privacy policy is provided: https://projectredcap.org/software/mobile-app/privacypolicy/
- Following this, there will be an interview about your experiences of accessing pediatric rehabilitation services, your preferences with the use of a remote robot technology to support pediatric rehab, and how the healthcare system can use the remote robot technology and communication in a way that would be more beneficial for your family.
- The interviewer will ask for your consent for audio recording the interview. The interview will not be video recorded. We will not audio record your name or any identifying information. The recording helps us keep an accurate record of what you said. We will not use the recording for any other purpose. You may ask to have the recording device turned off at any time, without giving a reason. The consent for audio recording is at the bottom of this form.
- Interviews may be virtual, taking place over video conference (Zoom), by phone or by Remote Presence Robot which is a medical device for virtual care, to allow for participation where in-person options are not feasible.
- One-on-one interviews should take approximately 30-60 minutes.
- Interviews by video conference will take place using Zoom, or Remote Presence Robot. The privacy policy for Zoom can be found here: <https://explore.zoom.us/docs/en-us/privacy.html>. There will be an option for phone in as well if that is your preference. Remote Presence Robot has a private sign-in for the researcher (at University of Saskatchewan), and for the hospital where the robot is located. It is not possible for other people to accidentally join this secured link.
- University of Saskatchewan’s agreement with Zoom ensures that all data will be routed through servers in Canada.
- Zoom implements safeguards to protect your personal information from accidental or unlawful use. The Internet, however, cannot be guaranteed to be 100% secure. Remote Presence Robot is only accessible to the two parties on either end of the robot. Uninvited guests cannot enter this secure link.
- If you require a translator, a translator will be provided to you at no cost. The translator will sign a confidentiality agreement.
- You cannot make any unauthorized recordings of the content of a meeting/data collection session.
- After your interview, you will be given the chance to review the typed copy (transcript) of your interview. You can add, change, or remove information from the transcript as you see fit. You will be given 1 week to provide changes to the transcript, otherwise it will be used in the form that it was sent to you. A transcript release form will be provided.
- Audio recordings will be transcribed using Otter.AI, an online application that transcribes speech to text. Otter.ai data is stored on servers in the United States, so will be subject to US regulations. The privacy policy for Otter.AI can be found here: <https://otter.ai/privacy-policy>
- Recordings will only be identified by an ID number to maintain your privacy and confidentiality.
- Please feel free to ask any questions regarding the procedures and goals of the study or your role.

**Funded by:**

- This research is funded by the Saskatchewan Health Research Foundation and Saskatchewan Council on Patient Oriented Research.
- The research team has no conflicts of interest to be declared.

**Potential Risks:**

- There is a chance that you might experience mental or emotional discomfort as we ask you to talk about your experiences.
- If you feel that support is needed, please feel free to let the research team know so that we can assist you to find appropriate support. You may also follow up with your family physician or counsellor, if appropriate and available. Additional sources of support include:
  - Professional Health Advice and Mental Health Support (8-1-1)
  - Crisis Services Canada – Suicide Prevention and Support (1-833-456-4566)
  - Prince Albert Mobile Crisis Unit (306-764-1011)
  - Saskatoon Mobile Crisis (306-933-6200)
  - Holistic Care Team, Deschaumbeault Lake Health Centre (306-632-2106)
  - Holistic Care Team, Arthur Morin Memorial Health Centre (306-758-2063)

**Potential Benefits:**

- Benefits to participating include the ability to share and provide information that may help in the provision of culturally appropriate pediatric rehabilitation services and children and youth health promotion amongst your community.

**Compensation:**

- Participants will be offered an honorarium of $50 for their participation, even if they withdraw their participation. Traditional Indigenous Healers, Indigenous Elders or Traditional Knowledge Keepers will receive an honorarium of $100 and gifts of tobacco or cloth as appropriate. Honorariums will be delivered through Canada Post (cheque) or via Direct Deposit to your bank or through direct e transfer. If payments to an Elder or Knowledge Keeper exceeds $100, the participants may need to provide their Social Insurance Number (SIN) to University of Saskatchewan Financial Services for taxation audit purposes.
- Any personal information collected as a record of honorarium payment will be stored separately from the data and kept for 5 years.

**Confidentiality:**

- The information we collect in this study will be shared through presentations, conferences, and journal publications.
- In all publications or presentations, your identity will be kept confidential. Data will be presented in a summarized form, with information from all participants combined. Direct quotations may be reported from interviews, but your name will never be used. All identifying information will be removed from the report.
- Please note that although we will make every effort to safeguard your data, we cannot guarantee the privacy of your data, due to the technical vulnerabilities present in any online video conferencing platforms.
- Our research team will conduct the videoconference in a private area of our home or office that will not be accessible to anyone outside of the research team. We recommend that you do the same.
- By signing this consent form, you are giving your permission to have the interview to be audio recorded. You will also be asked prior to the start of the interview to provide oral consent for audio recording. If you choose not to be recorded, we will keep hand written notes of the interview instead of a recording.

Please put a check mark on the corresponding line(s) to grant or deny your permission:

| I grant permission to be audio recorded |  |
| --- | --- |
| I grant permission to obtain my oral consent for audio recording |  |

**Storage of Data:**

- The principial investigator (PI), Stacey Lovo, will be responsible for the data storage. Data collected from paper files (i.e. consent forms and health questionnaires) will be kept in a secure/locked file cabinet in a locked office at the Deschaumbeault Lake Health Centre, or Arthur Morin Memorial Health Centre accessible by community research assistants only and transferred to PI Dr. Stacey Lovo at the earliest possible date. Paper files will be transported in a locked briefcase by Dr. Lovo to Saskatoon. Paper copies of consent forms will be stored in a locked cabinet, in the PI’s locked office at the University of Saskatchewan. Electronic information will be stored on the PI’s password-protected computer during data collection, and immediately transferred to Datastore and to One Drive for transfer to the Canadian Hub for Applied and Social Research (CHASR) at the University of Saskatchewan. No data will be stored using Zoom’s internal cloud storage.
- Data will be stored for the minimum required storage period of five years post-publication. The master-list that connects your name to your participant identification code will be kept for a 6-month period. This list will be stored separate from all other data in a secure electronic folder. It will be permanently deleted after a 6-month period.
- Once the data is no longer required and following the required storage period, the data will be destroyed beyond recovery.
- Identifying information, (e.g., Consent Forms, Master List) will be stored separately from the data collected.
- To protect your information, Zoom meetings will be secured with a password or a personal meeting room to prevent uninvited guests.
- Zoom audio recording will be used, but NOT saved to the cloud. It will be saved to the researcher’s laptop, transferred to Datastore and to Otter.AI application for transcription, and then final transcripts will be saved in Datastore. These are protected servers at the University and only the researchers will see the final documents. Audio recordings will be deleted off the laptop once saved to Datastore.
- Due to the unknown stability of internet connections, an audio recorder will also be used for Zoom and it will be used for Remote Presence Robot interviews as well. The audio recording will be deleted as soon as Zoom recording is verified has having worked. In the case that audio recording failed on Zoom recording, the data from the audio recorder will be transferred to Datastore and then Otter.AI and then final transcripts will be saved in Datastore. For meetings using the Remote Presence Robot, only the audio recorder will be used for audio recording. The data from the audio recorder will be transferred to Datastore and Otter.AI for transcription. Audio recordings will be deleted off the audio recorder once saved to Datastore.

**Right to Withdraw:**

- Your participation is voluntary and you can answer only those questions that you are comfortable with. You may withdraw from the research project for any reason, at any time without explanation or penalty of any sort.
- Your participation or non-participation will not affect your access to community services (such as health care or school), employment, or how you will be treated within the community.
- Should you wish to withdraw, please contact our research team using the contact information provided, and your data will be deleted from the research project and destroyed.
- Your information/data will not be shared with your treating health care practitioners; therefore, any current or future health care provided will not be affected by participating in this research.
- Your right to withdraw data from the study will apply until 2 weeks following your interview. After this, it is possible that some form of sharing the research findings will have already occurred and it may not be possible to withdraw your data.

**Follow up:**

- We will share findings from the study with all participants through e-mail or mail if you prefer regular mail a year following data collection.
- Research findings will also be posted on the Musculoskeletal Health and Access to Care website at University of Saskatchewan: <https://cchsa-ccssma.usask.ca/mhac/about/musculoskeletal-health-and-access-to-care.php>
- Findings are estimated to be available by one year following completion of the interview.

**Questions or Concerns:**

- Contact the researcher(s) using the information at the top of page 1.
- This research project has been approved on ethical grounds by the University of Saskatchewan Behavioural Research Ethics Board. Any questions regarding your rights as a participant may be addressed to that committee through the Research Ethics Office: [ethics.office@usask.ca](mailto:ethics.office@usask.ca); 306-966-2975; out of town participants may call toll free 1-888-966-2975.

**Preferred method of receiving documents (research findings, copy of signed consent form):

E-mail:** Please provide your e-mail address: **_____________________________________
OR
Mail:** Please provide your mailing address: **_______________________________________**

**Preferred method of providing consent:**

- Signed Consent
- Oral Consent

**Continued or On-going Consent:**

- If clarification or a second session with a participant is needed, this consent will be reviewed again to ensure ongoing consent.


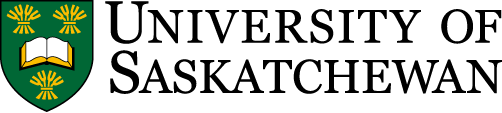


**CONSENT TO PARTICIPATE**

**Study Title:** Expanding Interprofessional Pediatric Rehabilitation in Northern Indigenous Communities with a Community-Directed, Team and Virtual Care Approach

- I have read (or someone has read to me) the information in this consent form.
- I understand the purpose and procedures and the possible risks and benefits of the study.
- I was given sufficient time to think about it.
- I had the opportunity to ask questions and have received satisfactory answers.
- I understand that I am free to withdraw from this study at any time for any reason and the decision to stop taking part will not affect my future relationships.
- I give permission to the use and disclosure of my de-identified information collected for the research purposes described in this form.
- I understand that by signing this document I do not waive any of my legal rights.
- I will be given a signed copy of this consent form.

I agree to participate in this study:

____________________________________ _______________________ ____________

Printed name of participant: Signature Date

____________________________________ _______________________ ____________

Printed name of legal guardian of participant: Signature Date

____________________________________ _______________________ ____________

Printed name of person obtaining consent: Signature Date

☐ I wish to be sent the results of the study

***A copy of this consent will be left with you, and a copy will be taken by the researcher.***

**Oral Consent:**

- In the event that this interview is conducted virtually (ie – via videoconference) you will be asked for your oral consent to be audio-recorded prior to the beginning of the interview.
- Or, if you do not feel comfortable providing a signature, consent for the whole process will be obtained orally, and the following section will be completed by our researcher.

I read and explained this consent form to the participant before receiving the participant’s consent, and the participant had knowledge of its contents and appeared to understand it.

|  |  |  |  |  |
| --- | --- | --- | --- | --- |
| *Name of Participant* |  | *Researcher’s Signature* |  | *Date* |
