# Supplementary material for: Developing an Interprofessional Pediatric Rehabilitation Model of Care in Northern Cree First Nation Communities: Protocol for a Needs Assessment and Codeveloped Intervention With a Qualitative and Participatory Action Approach
Source: JMIR Res Protoc. 2025 Sep 10;14:e69438. doi: 10.2196/69438 (PMC12461169; doi:10.2196/69438)
Supplement: Multimedia Appendix 2 [file resprot_v14i1e69438_app2.docx]

**APPENDIX I: COMMUNITY PARTICIPANT DEMOGRAPHIC AND GENERAL HEALTH**

**Note that a research team member will ask the questions to ensure appropriate language is used for complete understanding, with opportunity for explanation as needed. This survey will be available electronically for the research team member to complete with the patient family member; however, paper copies will be available as an option, hence the instruction on ensuring confidentiality.*

Thank you for agreeing to participate in this study. Please answer the following questions to the best of your knowledge. To ensure confidentiality, please do not put your child’s name on any of the following pages. If you have questions about the questionnaires, please speak to a member of the research team.

**ABOUT YOUR CHILD:**

1. Age: _______
2. Gender: Male Female Non binary Two spirit other: ________

**ABOUT YOUR CHILD’S BIRTH AND DEVELOPMENT:**

1. Did you or your child experience any complications during pregnancy, birth or infancy? Yes/ no (branching logic): If yes: Please check all that apply:

During pregnancy :

 gestational diabetes

 preeclampsia

 ecamplsia

 gestational hypertension

 placenta previa

 placenta abruption

 multiple gestation

 preterm labor

 depression

 infection

 anemia

 thyroid disorder

 intra-uterine growth

 Other: ___________

During childbirth :

 infection

 obstructed labor

 natural birth

 c- section

 low apgar

 low birth weight (please specify if known) _____

 Other: ________

In infancy:

 plagiocephaly

 torticolis

 ear infection

 respiratory infection

 newborn jaundice

 asthma or other breathing concerns

 Other: ________

1. Did you have any concerns about your child’s early development?

 Gross motor (ie – delayed with sitting, rolling over, crawling, etc)

 Speech/oral motor (ie – challenges with speech, eating, swallowing)

 Fine Motor (ie – holding a pen/scissors, drawing/writing, eating with ustensils, manipulating small objects)

 Cognitive (ie – short attention span, lack of curiosity, difficulty speaking, inability to remember things)

 Social-emotional (Ie – did your child interact with others and show emotion as expected for their age?)

 Behavioral (ie – prolonged tantrums, challenges with minor changes in routine, challenges with situations that are frustrating)

1. Please indicate which developmental skills are *currently* areas of concern for your child. Please provide any comments regarding details of your child's difficulty or your observations of your child's skills in these areas.

 Sitting  Bathing

 Standing  Eating
 Crawling  Dressing

 Walking  Toileting
 Sleeping  Writing
 Stair climbing  Vision

 Motor Skills  Social Skills

 Hearing  Attention

 Speech  Other _____________________________

 Cultural participation

Comments: _________________________________________

1. Please check/list specific activities that are hard because of the above difficulties

Meal time School Travel Interacting with family Playing with peers

Playing independently Shopping Vacations Sports Cultural activities

Other Comments: _____________________________________________________________

**ABOUT YOUR CHILD’S HEALTH HISTORY:**

1. Please check any of the following medical conditions that your child may have or

have had:

Headaches, Comments: _______________________________________________________

Developmental delay, Comments: ________________________________________________

Down Syndrome, Comments: ___________________________________________________

Heart problems, Comments: ___________________________________________________

Stomach or digestive problems, Comments: _______________________________________

Lung or breathing problems, Comments: __________________________________________

Seizures, Comments: __________________________________________________

Neurological (Stroke, Cerebral Palsy, Epilepsy), Comments: ___________________________

Hearing problems, Comments: ________________________________________________

Vision problems, Comments: _________________________________________________

Learning disability, Comments: __________________________________________________

Autism, Comments: _______________________________________________________

Other muscle, bone, or joint problems or pain. Please list where your child has muscle, bone or joint problems or pain: ________________________________________________________

_____________________________________________________________________________

Any other health issues: _____________________________________

1. Does your child currently receive any therapy services (for example: speech, occupational therapy, physical therapy) or have they received services in the past? Yes/No

If no, why not:  not needed  no referral  too far to travel  costs  on waiting list,  other___

If yes, which services:  speech language pathology  occupational therapy  physical thearpy  neuropsychology  other___

1. Is your child being followed by any specialists?

    No specialists.  Neurology  Orthopedics  Oncology  other___

**USE OF EQUIPMENT**

1. Does your child use any assistive or adapted equipment (such as a walking aid, hearing aid, or other)? Yes/No

If yes:  Walking aid  Hearing aid  Wheelchair  Lifts  other: List: _________

  If no: Do you think your child would benefit from having assistive or adapted equipment Yes/no
        If yes: What are the barriers to obtaining appropriate equipment?

 Not sure what would be helpful,  unable to use/obtain recommended equipment
 associated expenses.  Other: __________

**MORE ABOUT YOUR CHILD**

1. What do you believe are 3 things your child does well? (Please select the top 3 strengths) ________________________________________________________________________________________________________________________________________________________________________________________________________________________

 Caring/kind

 Cooperates

 Resilient
 Shares/takes turns and can compromise

 Is a good listener
 Asks for help when needed
 Has a good sense of humor

 Uses words to express needs/wants/ideas

 Participates in discussions/communication at home/school/with friends

 Good at solving puzzles

 Follows rules/routines well

 Stays focused on tasks
 Learns from mistakes and solves problems

 Creative/artistic
 Ability to use technology (ie – iPads)
 Plays sports or games (including video games)

 Dances, acts, sings, or plays a musical instrument

 Other

1. What are some of your child’s favorite activities and/or toys?

______________________________________________________________________________________________________________________________________________________________________________________________________________________________

1. Is there anything else you would like us to know about your child? __________________________________________________________________________________________________________________________________________________________________

**ABOUT YOUR FAMILY:**

1. Family & Household Members: Including you, how many people reside in your household? ____
2. Please select which household members currently reside with you?
    Children Spouse Grandparents Other adult relatives Other child relatives
    Other? List: ________
