# Supplementary material for: Developing an Interprofessional Pediatric Rehabilitation Model of Care in Northern Cree First Nation Communities: Protocol for a Needs Assessment and Codeveloped Intervention With a Qualitative and Participatory Action Approach
Source: JMIR Res Protoc. 2025 Sep 10;14:e69438. doi: 10.2196/69438 (PMC12461169; doi:10.2196/69438)
Supplement: Multimedia Appendix 3 [file resprot_v14i1e69438_app3.docx]

**Community Participant Semi-Structured Interview Guide**

**(Group and Individual)**

***Please note that these questions will be reviewed and further informed by our community partners… as they may have other suggested questions/modifications to questions***

**Preamble:**

Hello, my/our name(s) is/ are X and my pronouns are (she/he/they), and we are part of the research team looking at your community’s needs for pediatric rehabilitation services such as physical therapy, occupational therapy, or speech therapy. We are also interested in how the robot at the community health facility can be used to join pediatric rehab providers from other locations with your community members to help work with children and families.

Thank you for agreeing to talk to me/us about your experiences with accessing pediatric rehabilitation services.

As you know from the consent form we just reviewed together, the information you provide will help us to better understand: 1) your family’s experiences accessing pediatric rehabilitation services; 2) what your unique preferences might be regarding the use of technology, specifically a remote robot technology, to support children’s rehabilitation; 3) what type of information would help to understand if using robot technology is helpful in working with children and families in your community.

This information you provide will help us to better understand how the health system can work better for families and children who need to access rehabilitation services, which may also be relevant to other members in your community. What you share with us will help design and evaluate a process for using remote robot technology as well as in person care to work with children and families, with the hope of improving access to pediatric rehabilitation services.

We hope this information will be used to develop further health services and supports to benefit the community.

This interview/ discussion should take approximately 30 minutes for one-on-one interviews, and up to 2 hours for group discussions.

I/ We will be recording our conversation today and the recording will then be typed up into a written script for analysis after the research project is completed. Are you ok with proceeding with the interview at this time?

Do you have any questions before we get started?

*If no…*

Let's get started…I/we am/ are turning on the recorder now.

1. Tell me/ us a bit about your/your child’s health condition, movement or functional difficulty, and their needs for healthcare and rehabilitation services (*pause after this statement and wait for a response; and if needed, use the following bulleted list as probes to draw out information from the participant)*
   1. How long have you experienced this problem or problems?
   2. How has this affected your child’s and family’s life?
      1. Physical functioning (intense activities, moderate activities, dressing and bathing daily activities)?
      2. Social participation?( i.e. activities with family, friends, community, school)
      3. Emotional consequences? (feeling tense, depressed, having enough energy)
      4. Abilities to engage in and/or practice culture and/or spiritual activities?
      5. Other? (e.g. sleep)
2. What types of supports/ services have you found helpful for helping you/your child’s growth and development?
   1. Health care services? (such as physical, occupational, or speech therapy?)
   2. Community supports or programs?
   3. Local/traditional cultural practices?
   4. Others? (e.g. companion animals, other self-management strategies?)
3. What types of services and/ or supports do you think would help to support your family and children’s overall abilities to do the things you want to do?

(prompt…PT, OT, SLP, psychology…)

- 1. What is important to consider when developing new services:
     1. language and culture,
     2. culture and protocols in your community,
     3. that new providers should be mindful and aware of to ensure care provided is culturally appropriate and responsive to your needs and preferences?

1. Can you tell us about any challenges you have had in trying to access pediatric care within your home community?
   1. Wait times or availability? (lack of services)
   2. Financial/ costs?
   3. Cultural?
   4. Comfort interacting with health care professionals?
   5. Language or interpretation? (cultural languages, use of pronouns or reference to incorrect gender, etc).
   6. Mobility accessibility?
   7. Experiences with racism?
   8. Other?
2. Can you tell us more about the challenges associated with travelling to access services? Or other challenges with accessing care outside of your community?
   - 1. Such as, finding childcare, appropriate transportation, accommodations, weather barriers?
     2. Prompts from Q4
3. Do you think that using technology like the (remote presence) robot might help families in the community access better pediatric care?
   1. Why or why not?
   2. What might be some important ways to ensure that your culture and perspectives are respected and honored while using robot technology?
   3. What do you see as potential challenges to using robot technology to work with children and families in your community? (these could be anything including language or other cultural practices… as examples)
4. Do you think that linking community members, such as yourself, to children’s health care providers using remote technology and with the support of local health care providers would be helpful for families, children, and youth in the community?
   1. Why or why not?
   2. If technology could be used to increase access to care (such as physical, occupational, or speech therapy) to work with children and families in communities such as yours, what do you think is needed to ensure that the process and interactions with health care providers are meaningful to you?
5. How would we know if this type of service was helpful/effective for the community?
   1. Do you have some ideas on what types of measures would help capture the usefulness of using robot technology to increase access to children’s rehabilitation services?
   2. (Alternate phrasing if needed: after using the robot technology for rehab services, what effects on children or their families would tell us that the service was helpful?)
      1. Participant experiences/ stories?
      2. Better quality of life?
      3. Better movement/ mobility?
      4. More able to participate in social/ community activities?
      5. Less travel from the community?
      6. Others?
6. What do you see as being important for helping promote children and youth health*?* (For example, practicing culture and language, community programs, etc.)
   1. What ideas do you have to help promote health for children and youth in your community?
7. Is there anything else you would like to share with us about either your experiences with children’s health, health care access/ use or what type of new service using the robot or other supports in your community might be helpful?

Thanks so much for your time and your thoughts. Before we finish today, I would like to go back to the consent form briefly.

**For Group Discussion/sharing circles**: As stated in the consent form, we ask that you do not share the contents of the discussion we just had outside of the group as to respect the privacy and confidentiality of the participating members.

**For individual interviews**: Now that we have been through the interview and you know what you have shared with me, I just want to go back though the sections we checked off and see if you still consent in the same way as before we started. It’s perfectly OK to change your mind on any of this. [At this point confirm all the check box decisions with the participant]
