# Supplementary material for: Developing an Interprofessional Pediatric Rehabilitation Model of Care in Northern Cree First Nation Communities: Protocol for a Needs Assessment and Codeveloped Intervention With a Qualitative and Participatory Action Approach
Source: JMIR Res Protoc. 2025 Sep 10;14:e69438. doi: 10.2196/69438 (PMC12461169; doi:10.2196/69438)
Supplement: Multimedia Appendix 4 [file resprot_v14i1e69438_app4.docx]

**Healthcare provider/ manager semi-structured interview guide**

***Please note that these questions will be reviewed and further informed by our community partners… as they may have other suggested questions/modifications to questions.***

**Preamble:**

Hello, my/our name(s) is/ are X and my pronouns are (she/he/they), and we are part of the research team looking at your community’s needs for pediatric rehabilitation services such as physical therapy, occupational therapy, or speech therapy. We are also interested in how the robot at the community health facility can be used to join pediatric rehab providers from other locations with your community members to help work with children and families.

Thank you for agreeing to talk to me/us about and share your guidance and experiences.

As you know from the consent form we just reviewed together, the information you provide will help us to better understand: 1) your experiences of helping families and children who require access to pediatric rehabilitation services; 2) what your unique preferences might be regarding the use of technology, specifically a remote robot technology, to support children’s rehabilitation; 3) what type of information would help to understand if using robot technology is helpful in working with children and families in your community.

This information you provide will help us to better understand how the health system can work better for families and children who need to access rehabilitation services, which may also be relevant to other members in your community. What you share with us will help design and evaluate a process for using remote robot technology as well as in person care to work with children and families, with the hope of improving access to pediatric rehabilitation services.

Our hope is that this information will be used to develop further health services and supports to benefit the community.

This interview/ discussion should take approximately 30 min - 1 hour.

I/ We will be recording our conversation today and the recording will then be typed up into a written script for analysis after the research project is completed. Are you ok with proceeding with the interview at this time?

Do you have any questions before we get started?

*If no…*

Let's get started…I/we am/ are turning on the recorder now.

1. Tell me/ us a bit about what you have observed regarding children and families needing pediatric rehabilitation services in the community? (*pause after this statement and wait for a response; and if needed, use the following bulleted list as probes to draw out information from the participant)*
   1. What types of pediatric services are needed in the community? (i.e., physical therapy, speech therapy, occupational therapy, audiology, psychology, dentistry, etc)
   2. What are some of the types of diagnoses or conditions that affect children in the community? (i.e., autism, spina bifida, Cerebral palsy, developmental delays, hip dysplasia, orthopedic injuries, etc.)?
   3. How has the need for pediatric services affected the lives of people in your community?
      1. Physical abilities?
      2. Social participation? i.e. activities with family, friends, community, work
      3. Emotional consequences?
      4. The ability to practice culture and/or spiritual activities?
      5. Other?
2. What types of supports/ services do you think would be helpful for families and children in the community who require pediatric rehabilitation services? For promoting children and youth/ adolescent health?
   1. Health care services (including physical therapy, occupational therapy, speech therapy, etc.)?
   2. Community supports or programs?
   3. Local/traditional cultural practices?
   4. Others? (e.g. companion animals)
3. Can you tell us about any challenges families or people in the community have reported or experienced in trying to access pediatric rehabilitation services?
   1. Travel?
      1. Such as, finding childcare, appropriate transportation, accommodations, weather barriers?
   2. Wait times?
   3. Financial/ costs?
   4. Cultural?
   5. Comfort interacting with health care professionals?
   6. Language or interpretation? (cultural languages, use of pronouns or reference to incorrect gender, etc).
   7. Mobility accessibility?
   8. Experiences with racism?
   9. Other?
4. What types of services and/ or supports do you think would help to overcome these challenges?
   1. What are community supports and strengths in the community that would help to support families needing pediatric rehab services?
   2. What types of information, cultural understanding and communication is important for new providers to have and demonstrate, to ensure care is culturally responsive, appropriate and patient-centered?
5. Do you think that using technology like the (remote presence) robot might help families in the community access better pediatric care?
   1. Why or why not?
   2. What do you see as potential challenges to using robot technology to work with children and families in the community? (these could be anything including language or other cultural practices… as examples)
6. How would we know if a new type of service were relevant and useful for the community? What types of measurements would help capture this?
   1. Participant experiences/ stories?
   2. Better quality of life?
   3. Better movement/ mobility?
   4. More able to participate in social/ community activities?
   5. Less travel from the community?
   6. Others?
7. What Western functional outcome scales have you traditionally used to assess children’s health, growth and development? In your opinion, how effective has that process been in the past?
8. From your experiences, which types of functional or pain outcomes were the most preferred by Indigenous children and families?
9. Describe your experience communicating about health with families when using Western functional or pain outcome measures/scales.
10. In your opinion, is there anything we could do to improve these measures?
    1. Any considerations in using these measures with robot technology?
11. Do you have any additional concerns about your ability to understand children’s health, growth and development from an Indigenous patients’ or family’s perspective?
12. Is there anything else you would like to share with us about either your observations of families and children requiring pediatric rehab services, health care access/ use or what type of new service using the robot might be helpful?

Thanks so much for your time and your thoughts. Before we finish today, I would like to go back to the consent form briefly. Now that we have been through the interview and you know what you have shared with me, I just want to go back though the sections we checked off and see if you still consent in the same way as before we started. It’s perfectly OK to change your mind on any of this. [At this point confirm all the check box decisions with the participant]
